# Estimating willingness-to-pay for neonicotinoid-free plants: Incorporating pro-environmental behavior in hypothetical and non-hypothetical experiments

**DOI:** 10.1371/journal.pone.0251798

**Published:** 2021-05-20

**Authors:** Xuan Wei, Hayk Khachatryan, Alicia Rihn

**Affiliations:** 1 Food and Resource Economics Department and Mid-Florida Research and Education Center, University of Florida, Apopka, FL, United States of America; 2 Department of Agricultural and Resource Economics, University of Tennessee-Knoxville, Knoxville, TN, United States of America; University of Naples Federico II, ITALY

## Abstract

This study investigates the extent to which individuals’ perceptions and attitudes toward pesticides and pollinator related labeling influence their preferences for eco-labeled products. An incentive compatible second-price auction and a hypothetical discrete choice experiment were used to elicit individual preferences for ornamental plants grown with or without controversial (neonicotinoid) pesticides. Positive attitudes toward pollinators, neonicotinoid labeling regulations, and labeling of sustainable production methods were found to be significant predictors of individual choice behavior. Individuals with attitudes expressing concern for pollinators and agreement with mandatory labeling and disclosure of neonicotinoids, showed a stronger preference for neonicotinoid-free plants. Our results suggest that both hypothetical and non-hypothetical experiments are consistent in predicting the general direction of consumer preferences despite the elicitation mechanism. Implications for relevant stakeholders are discussed.

## Introduction

Experimental auctions and discrete choice experiments are the two most important mechanisms to elicit consumer preferences and willingness to pay (WTP) for new products (e.g., food labeling), when market data are not yet available. However, more and more evidence has surfaced, showing that individuals tend to overstate the amount they are willing to pay for a good in a hypothetical setting compared to situations when there is financial consequentiality (e.g., [1, 2]). In response to increased skepticism regarding incentive compatibility in hypothetical settings, incentivized, non-hypothetical experimental auctions and choice experiments have become popular and are frequently used by empirical economists in their field and laboratory experiments (e.g., [3–7]). While non-hypothetical experiments certainly mitigate hypothetical bias, they often suffer from the limitation of relatively small sample sizes or specific groups (e.g., university students, convenience samples, etc.) which may impact sample representativeness.

In this study, we use both hypothetical online discrete choice experiments (DCEs) and non-hypothetical laboratory experimental auctions to analyze consumers’ WTP for landscape plants, grown with or without neonicotinoid (also known as “neonic”) pesticides. As previously discussed, samples used for non-hypothetical experiments are often scrutinized for sample representativeness and hence generalizability. To address this concern, we recruited U.S. consumers nationwide to participate in the online DCE. We tested the comparability of the two samples. Specifically, both the online DCE and lab experiment used the same screening questions (discussed in the Study Design and Methodology section) and the demographic results were compared to U.S. gardener demographics [8]. Similarities across the data points suggest sample representativeness was achieved in the experiments.

Given the increased consumer interest and demand for environmentally friendly production practices, the second objective is to elicit consumers’ attitudes toward neonicotinoid pesticides and pollinators (in general), as well as explore the differences in consumers’ WTP for products with labels disclosing the absence or presence of neonicotinoids during production. Despite increased media attention to neonicotinoids’ potential adverse effects on pollinators, previous studies have shown that general public knowledge about neonicotinoid pesticides remains low [9–11]. We further investigate the extent to which consumers’ attitudes toward pollinator health and conservation, neonicotinoid labeling, and relevant regulations influence their preferences by exploring the differences in WTP between various attitude-based dichotomous groups. Exploring the relationship between consumers’ attitudes and behavioral outcomes has been a key question in pro-environmental behavior (PEB) literature [12–21]. However, the majority of literature addressing this topic focuses on consumption choices related to sustainable food labeling such as organic, which is frequently associated with reduced environmental impacts (e.g., [22–25]). Nonetheless, implications for PEB with food labeling, such as organic, are less clear-cut given that personal health concerns linked to potential residual pesticide exposure in non-organic products may also drive organic purchasing behaviors [15]. Addressing PEB while reducing other factors that may impact choice is challenging but may be achieved through using products or services with the main benefit of use being environmentally focused. For instance, neonicotinoid pesticide use in the ornamental plant industry has potential environmental implications but does not directly impact human health through consumption of the products (unlike organic food products).

Surprisingly, there are a minimal number of studies investigating non-food product preferences (such as ornamental plants) associated with sustainable production practices. Wollaeger et al. [11] discussed ornamental plants grown and labeled with different pest management practices. They concluded consumers valued “bee-friendly” the most, but likely discounted products labeled “neonicotinoid-free.” Khachatryan et al. [26] showed that the pollinator-friendly attribute along with other eco-labels (e.g., Certified Organic, Fresh from Florida) were positively associated with consumers’ preference for ornamental plants. While Wollaeger et al. [11] and Khachatryan et al. [26] considered the pollinator-friendly attribute in general (along with other sustainable production practices), Wei et al. [10, 27] focused specifically on neonicotinoid labeling. Using experimental auction data, they found consumers would pay a price premium for plants with labels disclosing the absence of neonicotinoids but did not differentiate information formats when a plant was grown with neonicotinoids. This paper and Wei et al. [10, 27] belong to the same larger research project, so the methods are complementary and part of the data related to the experimental auction are overlapping. However, in this paper, we are particularly interested in investigating the attitude-PEB relationship through exploring consumers’ attitudes and their consumption behavior toward pollinator-friendly products. Meanwhile, testing the comparability between the online DCE and experimental auction samples also serves as a check for sample representativeness and consistency of findings with Wei et al. [10, 27].

This study provides several contributions to the existing experimental economics literature. First, we demonstrate that small sample size and location specificity do not necessarily result in sample non-representativeness in laboratory or field experiments. Frequently, results drawn from laboratory and field experiments are questioned for generality and conclusiveness due to small sample sizes and concerns about sample representativeness. Using a topic with relatively low consumer awareness (i.e., neonicotinoids), we show consistency in subjects’ demographic characteristics and attitudes toward pollinators and neonicotinoids between two samples drawn from two subject pools (e.g., national and local).

Secondly, we confirmed the WTP estimates differ across DCEs and second-price auctions. Existing literature has shown (in most cases) non-hypothetical DCEs elicit higher WTP estimates than the incentive-compatible experimental auctions (e.g. [7, 28, 29]). Lusk and Schroeder [7] and Gracia et al. [29] further showed that WTP estimates in second and *n*-th price auctions are as much as two to three times lower than the valuations implied from the non-hypothetical DCEs. Gracia et al. [29] found WTP estimates may also be attribute dependent. They found in some cases, WTP for the transport animal welfare label from the experimental auction was larger than those of non-hypothetical DCE. In our case, the large WTP disparity between the two experiments were further amplified by the hypothetical nature of the online DCE (more than ten times). The WTP gap can be decomposed into two major sources: differences in the experimental methods (DCE vs. second-price auction) and differences in the elicitation methods (stated-preference/hypothetical vs. real/non-hypothetical). After controlling for comparability and consistency in sample demographics, attitudes, and the experimental design in terms of the number of tasks, the complexity of attributes, and attribute levels, our conjecture is that in this research the significant WTP gap may largely be attributed to hypothetical bias in addition to the elicitation methods.

Lastly, we contribute to the attitude-behavior relationship in existing PEB literature. We validated the relationship between consumer attitudes and non-food environmentally friendly consumption decisions. By demonstrating a strong relationship between consumers’ positive attitudes toward pollinator conservation, neonicotinoid labeling regulations, labeling content, and purchasing intentions of neonicotinoid-free plants, this manuscript provides further insights into the relationship between attributes and PEBs through a unique perspective on PEB-driven consumption. Investigating a topic (neonicotinoid-free plants) that is directly related to the environment and has limited other drivers (e.g., personal health benefits, etc.) provides an opportunity to focus on purely PEB-driven consumption patterns. To date, this research direction has yet to be explored.

## Theoretical background and motivation

From the perspective of demand models and incentive compatibility, consumer preferences for private goods using experimental auctions (e.g., second-price auctions) and DCEs should be the same. Theoretically, both second-price auctions and DCEs are demand revealing and should induce subjects to truthfully reveal their true preferences regardless of the elicitation technique [30]. However, Horowitz [31] proved that experimental auctions are not always theoretically incentive-compatible. Individual subjects may underbid with respect to the dominant strategy [32–34]. On the other hand, from a psychological or behavioral perspective, preferences revealed in experimental auctions and DCEs may diverge due to biases and errors in the decision-making process [35]. For example, the cognitive load (such as the number of choice tasks and number and complexity of attributes and attribute levels) may result in WTP discrepancies across DCEs in different studies [28].

The general agreement on the existence of WTP differences has motivated many studies to investigate why this occurs, how much the gap varies across different elicitation methods, and to compare WTPs between hypothetical and incentivized experiments [7, 28, 29, 35–37] (for a meta-analysis see [38, 39]). Particularly, Gracia et al. [29] found that differences in WTP between incentivized *n*-th price auctions and DCEs are not only associated with individuals’ demographic characteristics but are also attribute dependent. In contrast, Grebitus et al. [35] proposed that personality traits explain a significant portion of hypothetical bias in WTP estimates elicited from second-price auctions and DCEs. Given these variances, the inclusion of more than one study methodology serves to test the robustness and validity of the results. Incorporating multiple methods is particularly important when investigating a product or attribute that is less known in the marketplace (e.g., neonicotinoid-free plants).

In addition to methodological variances and differences based on standard demographic characteristics, there is increased recognition of the importance of psychological factors such as consumers’ perceptions and attitudes which affect choice decisions. This is a central component of PEB research [13–21]. Existing studies demonstrated consumer attitudes are important factors that drive PEB [12, 40–45]. For instance, Klӧckner [46] determined that consumers’ intentions to act in an environmentally friendly way were directly predicted by “attitudes, personal and social norms, and perceived behavioral control.” Several experiments highlight consumers’ environmental awareness and attitudes in the food industry positively impact their consumption choices where foods with reduced environmental impacts are preferred (e.g. [23, 24]). Studies addressing attitudes and PEB consumption choices that have implications for horticulture industry stakeholders are limited (e.g., [11]); yet, studies suggest similarities to the PEB observed in the food industry. For example, Carrico, Fraser and Bazuin [12] discussed that individual interests and social pressures positively predicted residential lawn fertilizer usage, while environmental concerns did not. The researchers suggested that homeowners’ decisions involve making tradeoffs between aesthetics, environment, and personal health concerns related to potential pesticide exposure. This suggests a similar pattern of attitude-behavior occurs in the horticulture industry, but it has not been thoroughly addressed. For instance, environmentally sensitive consumers may be more likely to take environmentalism into account and purchase plants with limited exposure to pesticides.

Environment-related attitudes are central to PEB research focusing on food products, yet studies addressing PEB in the horticulture industry are scarce. By incorporating attitude scales into consumer preference experiments, this is one of the first interdisciplinary studies incorporating the two branches of literature to investigate how participants’ attitudes influence their preferences for non-food consumption under different elicitation methods. Using neonicotinoids (a controversial type of systemic pesticide) in our experiments is of interest because neonicotinoids have been reported to have negative environmental impacts (i.e., harmful for pollinators) without consumption-related factors given the ornamental nature of the plants [47, 48]. Another potential benefit related to increasing demand for pollinator-friendly plants is that due to increasing urbanization, there is an opportunity to improve pollinator habitat with sustainable landscape practices. For instance, 90 million U.S. households (78% of all U.S. households) have yards, landscapes, or gardens that can be managed in a way to improve pollinator health [49, 50]. However, homeowners’ landscaping practices may be influenced by their knowledge and attitudes toward neonicotinoids and pollinators (in general). Thus, investigating the link between attitudes and PEB in the neonicotinoid and ornamental plant context helps provide information to encourage environmentally conscious consumption behavior among U.S. homeowners.

## Study design and methodology

This study was approved by the University of Florida Institutional Review Board (approval #201601783). The form of consenet was written. Two different experiments were administered to address the research questions, including an in-person experimental auction (non-hypothetical) and an online DCE (hypothetical). As aforementioned, the samples were recruited from two population pools. Online DCE participants were recruited nationwide through a professional survey platform, Qualtrics Inc. In contrast, participants in the experimental auction were recruited in Central Florida through flyers at local garden centers and advertisements on the research center’s social media platforms. Participants in both samples were screened and limited to individuals who were at least 18 years of age, lived in a home with a yard, and had purchased ornamental plants in the past 12 months. In addition, participants were required to sign an informed consent form before they could proceed to participate in the survey. Both experiments used the same survey questionnaire. For the online DCE, participants answered questions about purchase behaviors, pollinator related attitudes, and perceptions of neonicotinoid pesticides and pollinator insects before the choice experiments. Auction participants answered the purchase behavior questions before auctions and the attitudes and perception questions after the auctions. Both experiments concluded with questions collecting demographic information. Participants in the experimental auction received a 25-dollar incentive or equivalent (the winner would receive 25 dollars minus the market price, i.e., the second highest bid, and the winning product) to take part in the study. Participants in the online DCE did not receive any monetary incentive but received online reward points distributed by software platform and participant recruitment firm Qualtrics. To ensure the two samples were consistent and the results from the two experiments were comparable, statistical significance between the two samples was tested on participants’ observed demographic characteristics and attitudes toward neonicotinoid pesticides and pollinators. Task structures were highly consistent in both experiments. Non-price attributes and attribute levels were the same (Table 1). Subjects in the experimental auctions made 14 bids, while subjects in DCEs made 16 choices. The next section provides the sample statistics followed by a description of the major components of the study.

**Table 1:** Attributes and attribute levels used in the discrete choice experiment and experimental auction.

Attribute	Level 1	Level 2	Level 3	Level 4
Annual Bedding Plant Type	Impatiens (other)	Marigold	Pentas	---
Perennial Plant Type	Chrysanthemum	Dianthus	Salvia	---
Neonicotinoid Label	Neonicotinoid Free (text)	Bee Better Certified (logo)	Treated with Neonicotinoids	Protected from Problematic Pests by Neonicotinoids
		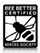		
Container Type	Conventional Plastic	Bio-degradable	---	---
Annual Bedding Plant Price^a^ (4-inch pot)	$1.15	$1.65	$2.49	$3.99
Perennial Plant Price^a^ (1-gallon pot)	$5.99	$6.99	$8.49	$10.49

Note

^a^ Price was determined based on local retail outlets’ prices (e.g., big box garden stores and independent garden centers) along with the USDA wholesale and retail price information [51, 52]. Price information was provided in the discrete choice experiments. Price was not an attribute in the experimental auction where participants bid the price they were willing to pay for each item in the auction experiment.

### Participants recruitment and sample characterization

Seventy-five participants were recruited from central Florida for the in-person experimental auction, and 420 respondents were recruited nationwide for the online DCE (Table 2). While the online sample had a relatively balanced male-to-female gender ratio (41%), the in-person Florida sample was more skewed toward females, with only 21% of the sample consisting of male participants. Even though subjects were drawn from two different population pools (Florida and the national population), the samples were reasonably consistent in their demographic characteristics and comparable with the exception of the aforementioned gender. Participants’ mean age was 54 years old (SD = 14.8 in the online sample and SD = 15.9 in the auction sample) in both experiments. Participants in the auction sample were slightly more educated and had higher median household incomes, but the difference was not statistically different between the two samples (Table 2). Participants reported a similar number of store visits, but online participants reported much higher spending on plant purchases than the auction sample. This is because Florida tends to have lower retail prices than the national average as a primary Southeast production state [52]. Compared to the general U.S. and Florida population statistics, our sample overrepresented older age individuals. However, the participants’ demographics are consistent with the core consumers for lawn and garden products who are 45 years old and older with the age group of 55 to 64-year-olds reported the highest spending on lawn and garden products [8]. Given the screening questions, this group was likely overrepresented in both the in-person and online samples.

**Table 2:** Descriptive summary statistics.

*Variables*	*Online*	*Experimental Auction*	*U*.*S*. *Population Estimates*^*a*^	*Florida Population Estimates*^*a*^
Number of participants	420	75	263,534,161^b^	17,719,854^b^
Male (%)	41%	21%**	49%	48.9%
Age				
Mean	54	54	-	-
Median	57	58.5	38.5	42.4
Ethnicity (%)				
White/Caucasian	87%	85%	75.0%	77.1%
African American	6.4%	5.8%	14.2%	17.6%
Hispanic	2.4%	4.3%	18.4%^c^	26.4%^c^
Asian	2.4%	1.4%	6.8%	3.7%
Native American	0.5%	0.0%	1.7%	0.8%
Pacific Islander	0.0%	0.7%	0.4%	0.2%
Other	1.4%	2.9%	5.5%	3.9%
Household income				
Mean	$62,000^d^	$62,800^d^	$92,324	$83,883
Median	$50,000^d^	$70,000^d^	$65,712	$59,227
Household size (mean)	2.9	2.6	2.6	2.65
Education level (%)				
High school / GED+	98%	100%	89%	88%
Bachelor’s degree+	40%	43%	33%	31%
Plant purchase behavior				
Number of visits (mean)	7	7	-	-
Amount spend per visit in USD (mean)	$70	$36**	-	-

Note

^a^ 2019 American Community Survey 1-year Estimates for U.S. and Florida population respectively (United States Census Bureau).

^b^ Estimates for population 16 years and over.

^c^ The category of Hispanic may be of any race and includes other race categories.

^d^ Household income is a categorical variable ranging 1–7. The corresponding income ranges for Categories 3 and 4 are $40,000-$59,999 and $60,000-$79,000, respectively. The online DCE sample has median 3 and mean 3.59 and the experimental auction sample has median 4 and mean 3.64.

** indicates significance between the variables of the two samples at the 5% significance level. Significance between continuous demographic characteristics (e.g., age, household size) between the two samples was determined using pairwise t-tests. Wilcoxon rank-sum test was used to determine the significance for the categorical variables (e.g., gender, ethnicity, education and income) between the two samples.

### Products and attributes

Products were selected based on the highest sales values reported in the 2014 USDA NASS Survey and included three annual bedding plants (Impatiens, Marigold, and Pentas) and three perennial plants (Dianthus, Chrysanthemum, and Salvia). Annual bedding plants were in 4-inch containers, while the perennial plants were in 1-gallon containers which coincides with common sizes available in the marketplace. The plants were differentiated by two types of environment-related attributes: labels communicating the absence or presence of neonicotinoids during production and labels describing the container types (biodegradable vs. conventional plastic). Four types of neonicotinoid labels were included. *Treated with Neonicotinoids* and *Protected from Problematic Pests by Neonicotinoids* (which is phrasing currently used by a major U.S. gardening supply retailer) were used to communicate the presence of neonicotinoids. Meanwhile, *Neonicotinoid Free* (text) and *Bee Better Certified*^*TM*^ (logo) were used to communicate the absence of neonicotinoids during production (Table 1). The *Bee Better Certified*^*TM*^ logo was developed by an international nonprofit organization (the Xerces Society for Invertebrate Conservation) to promote pollinator conservation in agriculture. To become *Bee Better Certified*^*TM*^ producers must commit to providing pollinator habitat while mitigating negative impacts from pesticides, including not using neonicotinoids (detailed information about *Bee Better Certified*^*TM*^ Production Standards is available at https://beebettercertified.org/docs). The experimental auction did not contain price information (explained shortly), but the DCE had four price levels per annual/perennial plants ($1.15, $1.65, $2.49 and $3.99 for the annual bedding plants; $5.99, $6.99, $8.49 and $10.49 for the perennial plants). Reference price points used in the choice experiment were based on retail observations in central Florida along with the USDA wholesale and retail price information [51, 52].

A fractional factorial design was designed in JMP Pro 13 to reduce the number of choices/choice scenarios in the experimental auction and DCEs. For the experimental auction, 14 annual bedding plants (with D-efficiency of 93.42) and 14 perennial plants (with D-efficiency of 92.25) with various combinations of attributes were used. For the online DCE, two blocks of 16 choice scenarios were separately constructed for the annual bedding plants (with a D-efficiency of 95.04) and the perennial plants (with a D-efficiency of 95.54) with each block consisting of eight choice scenarios.

### Experimental auction

**Fig 1:**
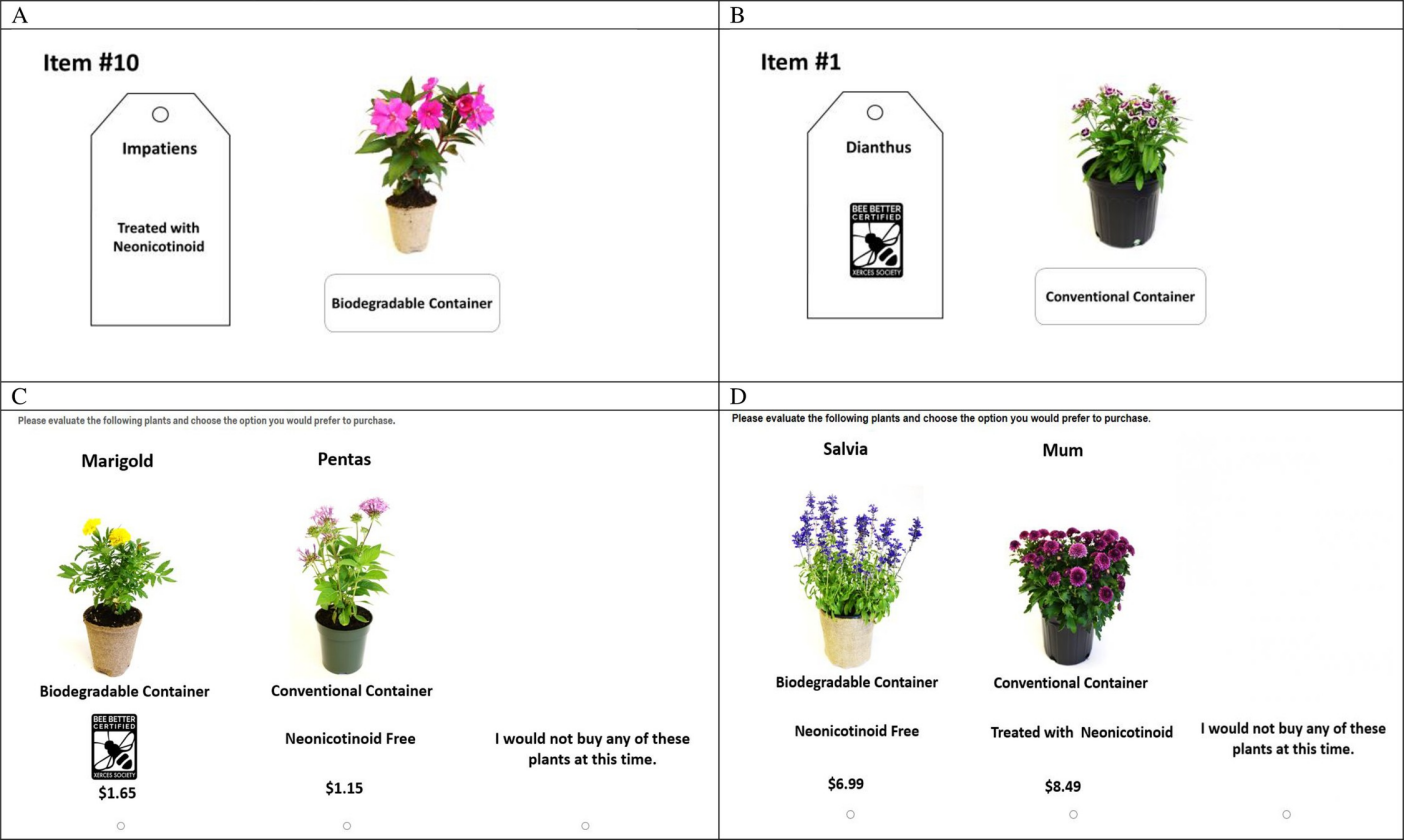
Representive examples of the experimental products. (A) Example of annual bedding plant auction item, (B) example of perennial plant auction item, (C) example of annual bedding plants choice set, (D) example of perennial plants choice.

Prior to the experimental auction study, 75 participants were randomly allocated into 15 sessions depending on their sign-up schedules. Upon arrival, participants signed an informed consent agreement. They were then briefed on the experimental procedures for a second-price auction (hereafter “SPA”). In each session, participants viewed images of the plants displayed on a computer monitor, while simultaneously submitting their bids. Each participant bid on 14 annual bedding plants and 14 perennial plants (see A and B in Fig 1 for experimental auction scenario examples) differentiated by their production practices (e.g., whether or not neonicotinoids were used). The SPA was non-hypothetical. At the end of each session, one annual and one perennial plant were randomly selected as the “winning items”. The auction winner (i.e., the person who bid the highest price) in each session paid the second-highest price and received the product. This method provides participants with an incentive to reveal their true WTP [53–57]. Specifically, if a participant bid lower than his/her true WTP, s/he risked forgoing a desirable purchase, while if s/he bid higher, s/he risked paying a price above the product’s perceived value. A utility-maximizing participant in the auction should submit a bid equal to his/her true WTP (i.e., truth telling is a weakly dominant strategy).

### Discrete choice experiment

In the online DCE, survey respondents were randomly assigned to view one block of eight annual bedding plants and one block of eight perennial plants after they agreed to the informed consent form. As a reminder, there were two blocks of eight annual and eight perennial plants. In total, participants made 16 hypothetical purchase choices. In each choice scenario, survey respondents selected the product they preferred from one of three options: plant A, plant B, or an opt-out option (i.e., I would not buy any of these plants).

### Attitude-behavior association and measurements of attitudes

The same survey instrument accompanied the two experiments to collect additional information on participants’ demographics, plant purchasing behaviors, and attitudes and perceptions about neonicotinoid pesticides, pollinators, and labeling practices preferences. In PEB literature, it is common to use well-established scales, such as New Environmental Paradigm (NEP) scale [58, 59] or environmental knowledge scale [60], to construct attitude measures. Meanwhile, as the importance of attitude-PEB association has been increasingly recognized, these traditional measures have been supplanted and/or updated by newly developed instruments seeking to measure for example, “ecological consciousness” [61] and “anthropocentrism versus ecocentrism” [62]. Accordingly, we also developed scales to meet the needs of this specific study even though these general scales might have some correlation with individual attitudes toward pollinators and neonicotinoid pesticides. Scales were developed to address three broad attitude categories, including: i) attitude toward neonicotinoid pesticides and pollinators, ii) attitude toward regulation of labeling neonicotinoids, and iii) attitude toward the importance of disclosing production methods on the label. These scales were built based on our previous research, and were discussed with the local ornamental horticulture industry experts [63–66].

A full list of the questions and statements that were used to measure attitudes are available in Table A1 in S1 Appendix. For example, the individual’s attitude toward neonicotinoid pesticides and pollinators was assessed using six statements related to that topic, including “I am concerned about the effects of neonicotinoid pesticides on pollinators.” For each statement participants indicated their agreement or disagreement on a 1–7 rating scale where response options were “strongly disagree = 1,” “disagree = 2,” “slightly disagree = 3,” “neither agree nor disagree = 4,” “slightly agree = 5,” “agree = 6,” and “strongly agree = 7”. Attitudes toward the regulation of labeling neonicotinoids were assessed by three statements. For example, using the same rating scale (1–7), participants indicated their level of agreement with “The federal government should require mandatory labeling of plants that are treated with neonicotinoid pesticides.” Their attitudes toward the importance of information disclosed on labels was based on their ratings of importance for five sustainable production practices. Specifically, participants rated the importance of labels (using a 7-point Likert scale with 1 indicating very unimportant and 7 very important) disclosing the following production method information: a) pesticide free, b) neonicotinoid free; c) non-GMO/GMO free, d) certified organic, and e) organically produced. It is worth noting that we purposely distinguished “certified organic” and “organically produced” because they may be viewed differently by participants. “Certified organic” was described as “the plants are certified as organically produced” by the USDA. On the other hand, “Organically produced” was described as “the plants are produced in an organic manner but are not certified organic.” Plants were mainly produced without using chemical substances (no chemical pesticides, no commercial/chemical fertilizers, no chemical herbicides, no preventative spraying).

From the complete list of statements and importance ratings, corrected item-total correlations (Table A1 in S1 Appendix) and principal components analysis (Table A2 in S1 Appendix) were used to refine the most relevant items and construct metrics quantifying participants’ attitudes. The results are shown in Table 3. Participants’ attitudes toward neonicotinoid pesticides and pollinators were measured using four statements. Considering equal weights of the four statements, the means of the four statements (*M* = 5.37, *SD* = 1.06 for the DCE, *M* = 5.19, *SD* = 1.11 for the SPA) were used to measure the participant’s overall attitude toward neonicotinoids and pollinators. The Cronbach’s *α* is 0.66 for the online DCE sample, slightly lower than the SPA sample (*α* = 0.79), but both are deemed as an acceptable level of reliability according to Ursachi et al. [67]. Participants’ attitudes toward regulations of labeling neonicotinoids were measured using two statements (*α* = 0.84, *M* = 5.99, *SD* = 1.26 for the DCE, *α* = 0.92, M = 5.93, SD = 1.30 for the SPA). Lastly, five importance ratings were used to measure participants’ attitudes toward the importance of information disclosed on labels (*α* = 0.89, *M* = 5.05, *SD* = 1.41 for the DCE, *α* = 0.90, M = 5.30, SD = 1.30 for the SPA). The Wilcoxon rank-sum test statistic indicates that participants’ attitudes toward pollinators, neonicotinoid labeling regulations, and labeling sustainable production methods are fairly consistent between the two samples. Participants in the online DCE sample were a little inconsistent as they indicated slightly more concern about the effects of neonicotinoids on pollinators but perceived disclosing producing without neonicotinoids as less important than the SPA participants. Online participants may view other factors that were not included in this study as having a great impact (e.g., Colony Collapse Disorder, monoculture agriculture, loss of habitat, etc.). It may be also caused by regional differences. What was viewed as important to Florida participants may not be a national trend.

**Table 3:** Descriptive statistics for attitude scale constructs.

	Online DCE	SPA
	Mean (Std. Dev.)	Mean (Std. Dev.)
Attitude toward neonicotinoid pesticides and pollinators^a^		
I am concerned about the effects of neonicotinoid pesticides on pollinators.	5.48 (1.45)	4.89** (1.52)
We may face a pollination crisis where crop yields decrease because of fewer pollinator insects.	5.73 (1.43)	5.69 (1.41)
Pollination is vitally important to terrestrial ecosystems and to crop production.	6.39 (1.15)	6.26 (1.05)
I would be willing to accept an increase in my annual taxes of $100 next year to promote neonicotinoid-free pesticides.	3.98 (2.02)	3.83 (1.81)
**Mean**	**5.37 (1.06)**	**5.19 (1.11)**
**Cronbach’s *α***	**0.66**	**0.79**
Attitude toward labeling neonicotinoids^a^		
The federal government should require mandatory labeling of plants that are treated with neonicotinoid pesticides.	5.86 (1.46)	5.85 (1.42)
Neonicotinoid labeling should be mandatory, because consumers have a right to be informed.	6.11 (1.24)	5.97 (1.31)
**Mean**	**5.99 (1.26)**	**5.93 (1.30)**
**Cronbach’s *α***	**0.84**	**0.92**
Attitude toward the importance of production method disclosed on a label^b^		
Pesticide free	5.70 (1.67)	5.84 (1.41)
Neonicotinoid free	4.97 (1.60)	5.42** (1.55)
Non-GMO/GMO free	4.83 (1.79)	4.79 (1.78)
Certified Organic^c^	4.87 (1.75)	5.30 (1.51)
Organically produced^d^	4.89 (1.69)	5.22 (1.54)
**Mean**	**5.05 (1.41)**	**5.30 (1.30)**
**Cronbach’s *α***	**0.89**	**0.90**

^a^ Participants indicated their level of agreement with the statements using a 7-point Likert scale where 1 = strongly disagree and 7 = strongly agree.

^b^ Participants indicated the level of importance of the information disclosed on the label using a 7-point Likert scale where 1 = very unimportant and 7 = very important.

^c^ “Certified organic” was described as “the plants are certified as organically produced” by the USDA.

^d^ “Organically produced” was described as “the plants are produced in an organic manner but are not certified organic.” Plants were mainly produced without using chemical substances (no chemical pesticides, no commercial/chemical fertilizers, no chemical herbicides preventative spraying).

** indicates significance between the variables of the two samples are statistically significant at the 5% significance level. Significance between attitude scales in the two samples was determined using Wilcoxon rank-sum test.

From these three attitude scales, four dichotomous groups were generated. Specifically, using the three attitude scales (i.e., neonicotinoids and pollinators, regulation of labeling neonicotinoids, and importance of disclosing production methods on the label), participants were categorized into an agree or disagree group for the first two scales, and an important or unimportant group based on the importance of information on the label. Participants were categorized into the agree or important groups if their average rating across the relevant statements/items was no less (≥) than the mean rating. For example, when considering participants’ attitude toward pollinators, participants were categorized into the agreement group if their average rating across the four items was greater than the mean attitude measure (i.e., ≥ 5.37) and categorized in the disagreement group if their average rating was less than the mean (i.e., <5.37).

Further, the fourth dichotomous group was used to separate participants based on an overall PEB attitude score (Table 4). Participants’ overall attitudes were regarded as PEB if all three individual attitude measures were equal to or larger than their corresponding sample mean. Conversely, if any of the three individual attitude measures were smaller than the corresponding sample mean, participants’ overall PEB attitudes were regarded as Non-PEB. In order to measure the internal relationship among the three attitude constructs and the overall PEB measure, we computed Pearson’s correlation coefficients for the three individual attitude measures and the overall PEB attitude measure (Table 4). Pearson’s correlation coefficients among all attitude scales exhibited a strong, positive correlation for the online DCE sample. For the SPA sample, correlation coefficients between Category 1 (i.e., attitude toward neonicotinoid pesticides and pollinators) and the other two categories were slightly below 0.5, which were deemed acceptable.

**Table 4:** Correlation matrix of the attitude measures.

	Online DCE				SPA			
Variables	1	2	3	4	1	2	3	4
1. Attitude toward neonicotinoid pesticides and pollinators	-				-			
2. Attitude toward labeling neonicotinoids	0.59	-			0.41	-		
3. Attitude toward importance of disclosing production method on the label	0.62	0.90	-		0.41	0.93	-	
4. Overall PEB attitude	0.88	0.75	0.68	-	0.78	0.70	0.65	-

After the four dichotomous groups were defined, WTP estimates were computed for each group. The WTP estimates were then compared (in the data analysis section) to assess i) how they differed across the two elicitation methods and ii) deviated across the dichotomy groups.

### Data analysis

Participants’ bids in the SPA study are summarized in Table 5. There were 216 zero bids (distributed across all 28 plants), accounting for about 10% of the total 2,100 bids. The average bid was 2.57 dollars (SD = 2.07) for the 14 annual bedding plants (in 4-inch containers) and 3.86 dollars (SD = 2.62) for the 14 perennial plants (in 1-gallon containers). For the DCE, after excluding 33% of the choices due to the selection of the opt-out option (i.e., those who chose the “I would not buy any of these plants” option), the average price of the chosen item in the online DCE was 2.11 dollars (SD = 0.98) for the annual bedding plants across the 16 choice scenarios and 7.45 dollars (SD = 1.47) for the perennial plants across the 16 choice scenarios. Despite the differences in experimental methods, the bids (in the SPAs) and prices of the chosen item (for the DCE) for the annual bedding plants were consistent regardless of experimental method. In contrast, slightly larger discrepancies were observed between the bids and prices for the perennial plants.

**Table 5:** Summary statistics of the bid and price attributes.

	Experimental Auction Bid Value^a^	Online Choice Experiment Price Attribute^b^
Annual		
Minimum	$0.05	$1.15
Mean	$2.57 (SD = 2.07)	$2.11 (SD = 0.98)^c^
Medium	$2.00	$1.65
Maximum	$13.40	$3.99
Perennial		
Minimum	$0.25	$5.99
Mean	$3.86 (SD = 2.62)	$7.45 (SD = 1.47)^c^
Medium	$3.50	$6.99
Maximum	$16.00	$10.49

Notes

^a^ Zero bids were excluded from consideration in this comparison.

^b^ Reference price points used in the choice experiment were based on retail observations in central Florida along with the USDA wholesale and retail price information [51, 52].

^c^ Mean was calculated across chosen prices of Plant A or B options in each choice scenario. Observations (choosing the opt-out option) were excluded from the calculations.

A regression analysis was conducted to facilitate the comparison of WTP estimates for the neonicotinoid labeling attributes across the different attitude groups. The auction data was analyzed using random effects tobit models [68], while the online DCE data was analyzed using mixed logit models [69] in STATA MP13.1. The tobit model was introduce by Tobin [68] and is widely applied to analyze experimental auction data (e.g., [53, 55–57]). We assume that a latent variable bid* exists and represents the participants’ true WTP for the product. The latent variable is related to the observed bids as follows:

$${bid}_{ij}=\left\{ \begin{array}{c} \begin{array}{cc} 0 & if\;{bid}_{ij}^{*}\leq 0 \end{array}\\ \begin{array}{cc} x_{ij}\beta +c_{i}+u_{ij} & if\;{bid}_{ij}^{*}>0 \end{array} \end{array},\right.$$ (1)

where *bid*_*ij*_ is the auction bid of consumer *i* for an ornamental plant *j*. ***x***_***ij***_ is a vector of plant attributes and individual characteristics that influence the consumer’s bidding price, *c*_*i*_ is the between-individual error term (i.e., individual-specific random effects varying across each individual *i* but not plant *j*) and *u*_*ij*_ is the within-individual error term, which has a normal distribution with a zero mean and variance $\sigma_{u}^{2}$. Therefore, the mean WTP (i.e., *E*(*bid*|*x*_*ij*_)) for a unit change in variable *x*_*ij*_ can be consistently estimated as

$${WTP}_{x}=N^{-1}\sum_{i=1}^{N}\left\{\Phi [\left(x_{ij}\hat{\beta }+c\right)/{\hat{\sigma }}_{u}]\right\}.$$ (2)

On the other hand, the mixed logit was developed by McFadden and Train [69] is commonly used by empirical researchers in DCE data analysis (e.g., [70–74]). Using the Random Utility Model (RUM) approach, the utility associated an individual *i* choosing alternative *j* in choice scenario *t* can be written as

$$U_{ijt}=\beta_{price}{price}_{ijt}{+x}_{ijt}\beta_{x}+\varepsilon_{ijt},$$ (3)

where *β*_*price*_ indicates the coefficient for the price of a plant in each option, and ***β***_***x***_ is a vector of unknown parameters to be estimated for other attributes such as plant type, container type and different types of neonic labels. *ε*_*ijt*_ is assumed to be independent and identically distributed with type I extreme value distribution. Therefore, in the mixed logit model, the choice probability that individual *i* would choose alternative *j* in choice scenario *t* can be expressed as:

$$P\left(\beta_{i}\right)=\int \left(\frac{\exp\left(\beta_{\mathrm{price}}{\mathrm{price}}_{ijt}+\beta_{ix}^{\prime }x_{ijt}\right)}{\sum_{j=1}^{J}\exp\left(\beta_{\mathrm{price}}{\mathrm{price}}_{ijt}+\beta_{ix}^{\prime }x_{ijt}\right)}\right)\phi \left(\beta_{ix}^{\prime }|\theta \right)d\beta_{x},\mathrm{for}\;j=1,\ldots ,J.$$ (4)

With repeated choices, we follow Revelt and Train [75] and compute the following probability of individual *i*’s observed sequence of choices as the product of standard logit formula (4) conditional on *β*_*i*_,

$$L_{i}(\beta_{i})=\prod_{s}P_{ij(i,t)t}(\beta_{i})$$ (5)

where *j*(*i*,*t*) is the alternative that person *i* chooses in scenario *t*.

Then the unconditional probability for the sequence of choices is

$$L_{i}(\theta^{*})=\int P_{i}(\beta_{i})f(\beta_{i}|\theta^{*})d\beta_{i}$$ (6)

where *f*(*β*_*i*_|θ*) is the density of *β*_*i*_ with parameter θ*.

Based on the estimated coefficients, the mean WTP value for a unit change in attribute can be calculated as the negative ratio of the mean estimated coefficient associated with the attribute and the price coefficient

$${WTP}_{x}=-\frac{\beta_{x}}{\beta_{price}}.$$ (7)

## WTP estimates results

The comparison of WTP estimates between the two different experiments and across the different attitude groups is summarized in Table 6. In general, the regression results show that participants are willing to pay a higher price for neonicotinoid-free plants and the neonicotinoid-free logo (i.e., “*Bee Better Certified*^*TM*^” logo) is the most preferred option regardless of the experimental method. Using “*Protected from Problematic Pests by Neonicotinoids*” as a base group, the estimated WTP for participants in the experimental auction is about 25 cents more for plants labeled with the “*Neonicotinoid Free*” text and 50 cents more for plants labeled with the “*Bee Better Certified*^*TM*^” logo. Participants in the online DCE are willing to pay 2.93 dollars more for plants labeled with the “*Neonicotinoid Free*” text, but as high as 6 dollars more for plants with the “*Bee Better Certified*^*TM*^” logo, indicating a stronger preference for the logo disclosing the absence of neonicotinoids. Conversely, when observing labels disclosing the presence of neonicotinoids (i.e., *Treated with neonicotinoid*) the auction participants’ WTP bids are not impacted (the coefficient is -0.10, but not statistically significant), but the online DCE participants’ WTP is reduced 1.20 dollars when compared to the base group. Comparing WTP across the two experiments methods, we identified a significant WTP gap. In contrast to [7] who found that non-hypothetial SPA bids were about two times lower than the valuations implied from the real DCE, the WTP estimate disparity in our non-hypothetical and hypothetical setting is much larger.

**Table 6:** Willingness-to-pay estimates for important product attributes, by dichotomous groups.

	Online Choice Experiment			Experimental Auction		
	WTP (Mean)		SE	WTP (Mean)		SE
Sample Total						
Neonicotinoid-free text label	2.932	***	0.263	0.250	**	0.079
Neonicotinoid-free logo	6.044	***	0.399	0.489	***	0.087
Neonicotinoid-treated text label	-1.204	***	0.244	-0.099		0.085
Attitude toward pollinator: Agree Group						
Neonicotinoid-free text label	6.815	***	0.781	0.516	***	0.122
Neonicotinoid-free logo	12.271	***	1.291	0.837	***	0.136
Neonicotinoid-treated text label	-0.815	*	0.389	-0.075		0.128
Attitude toward pollinator: Disagree Group						
Neonicotinoid -free label	1.351	***	0.246	-0.091		0.092
Neonicotinoid -free logo	3.721	***	0.336	0.051		0.099
Neonicotinoid -treated text label	-1.009	***	0.225	-0.135		0.100
Attitude toward neonicotinoid regulation: Agree Group						
Neonicotinoid -free label	5.224	***	0.541	0.512	***	0.115
Neonicotinoid -free logo	8.512	***	0.799	0.740	***	0.128
Neonicotinoid -treated text label	-1.650	***	0.427	0.101		0.120
Attitude toward neonicotinoid regulation: Disagree Group						
Neonicotinoid -free label	0.894	***	0.237	-0.177	*	0.089
Neonicotinoid -free logo	3.049	***	0.341	0.094		0.097
Neonicotinoid -treated text label	-0.511	*	0.239	-0.095		0.098
Attitude toward labeling importance: Important Group						
Neonicotinoid -free label	7.493	***	0.991	0.415	***	0.144
Neonicotinoid -free logo	13.155	***	1.624	0.740	***	0.158
Neonicotinoid -treated text label	-1.134	*	0.475	-0.096		0.154
Attitude toward labeling importance: Unimportant Group						
Neonicotinoid -free label	0.486		0.270	-0.173		0.095
Neonicotinoid -free logo	3.276	***	0.379	0.060		0.103
Neonicotinoid -treated text label	-0.858	***	0.252	-0.090		0.104
Overall PEB attitude: PEB Group						
Neonicotinoid -free label	9.657	***	1.494	0.750	***	0.205
Neonicotinoid -free logo	11.532	***	1.731	1.150	***	0.226
Neonicotinoid -treated text label	-0.952		0.547	-0.067		0.218
Overall PEB attitude: Non-PEB Group						
Neonicotinoid -free label	2.003	***	0.245	0.053		0.074
Neonicotinoid -free logo	5.048	***	0.375	0.233	**	0.081
Neonicotinoid -treated text label	-1.209		0.242	-0.115		0.080

Notes: Willingness-to-pay (WTP) estimates for online DCE sample were calculated based on coefficients from the mixed logit model. WTP estimates for the experimental auction sample were calculated using a tobit model.

^*^p<0.*05*.

^**^< 0.*01*.

^***^< 0.*001*.

The differentiated impact between attitude-based dichotomous groups on WTP for neonicotinoid labeling is confirmed in this study. On average, participants with positive attitudes toward pollinator conservation, neonicotinoid labeling regulations, and labeling content, as well as stronger overall PEB attitudes, are willing to pay higher price premiums for plants with labels indicating the absence of neonicotinoids relative to their counterparts. Online DCE participants in the PEB group are willing to pay 9.66 dollars more for plants labeled with the “*Neonicotinoid Free*” text and 11.53 dollars more for plants labeled with the “*Bee Better Certified*^*TM*^” logo. Auction participants in the PEB group are willingness to 75 cents more for plants labeled with the “*Neonicotinoid Free*” text and 1.15 dollars more for plants labeled with the “*Bee Better Certified*^*TM*^” logo. On the other hand, online DCE participants in the non-PEB group are only willing to pay 2 dollars and 5 dollars more for plants labeled with the neonicotinoid free text and logo, respectively.

## Discussion and conclusions

Concerning the potential impact of neonicotinoids on pollinator health, pro-environmental groups have encouraged retailers to clearly label products treated with neonicotinoids (US-EPA, 2013). However, public awareness of neonicotinoids among consumers remains low [9–11] and the extent to which products treated with neonicotinoids will impact consumers’ preferences is unknown. Regardless of low public awareness, research has indicated that consumers value pollinator conservation efforts [76–78]. The low consumer awareness of neonicotinoids in a pro-pollinator market provides a unique opportunity to assess how consumers’ existing (yet frequently uninformed) attitudes influence their WTP with minimal external factors since pollinator-related attributes do not directly impact human health (unlike organic food product studies). Given the low public awareness of neonicotinoids, the SPA results with a small sample size are regarded more susceptible to sampling bias and more likely to be questioned. By showing the comparability between the local and national samples, we provide evidence that the representativeness of a small sample can be improved through participant screening and recruitment even for a less-known topic. The reliability and generalizability of results derived from laboratory experiments with few data points should not be solely judged based on a small sample size.

Consistent with previous studies examining the external validity of hypothetical experiments (e.g. [36, 79]), our results suggest that both hypothetical and non-hypothetical experiments are consistent in predicting the general direction of consumer preferences despite differences in the elicitation mechanism. We also show WTP estimates differ significantly across DCEs and SPAs, with DCEs providing higher premiums for labels disclosing the absence of neonicotinoids regardless of the experimental setting. Previous studies investigating the inconsistency of WTP estimates across incentive compatible auction- and choice-based approaches found marginal WTP estimates from the DCE samples were about 2–3 times larger than those from the experimental auctions [7, 29]. However, the WTP estimates in our online DCE were further amplified due to the hypothetical nature of the mechanism (about 10 times larger). Our results suggest that differences in WTP estimates could mainly be driven by the hypothetical setting of the experiments in addition to the differences between experimental methods (DCE vs. SPA). Nonetheless, it is also possible that subjects in SPA were not incentive compatible and underbid the plants.

Our results also confirm the existence of a positive relationship between attitudes and an individual’s PEB (i.e., purchasing pollinator friendly ornamental plants). For participants with positive attitudes toward pollinators, they are generally willing to pay higher prices for plants that are not treated with neonic pesticides. This result is in line with other empirical studies which show that attitude is positively linked with environmentally friendly product consumption decisions, such as organic foods [80, 81] and environmentally friendly products in general [82, 83]. However, this relationship is weakened in the non-hypothetical SPA where real money is exchanged. The online survey respondents in a hypothetical purchase setting indicate much higher WTP estimates for ornamental plants labeled with the neonicotinoid-free text or logo compared to auction participants who were obligated to purchase a plant if they won the auction. Because of this non-binding condition, participants in the online DCE experiment could have over-stated the importance of not using neonicotinoids in their choices. This tendency is particularly evident for the positive attitude group in the online study.

There are several limitations to this study. Even though SPAs are incentivized, participants’ WTP may still deviate from their true preferences. Many empirical studies have shown that auction subjects tend to underbid with respect to the dominant strategy (see for example, [28, 33, 34, 84, 85]). Horowitz [31] proved that SPAs are not incentive compatible when subjects have non-standard expected utility functions. Participants may also fail to identify the optimal strategy and bid accordingly due to the experimental design [28]. Additionally, actual retail data from real retail settings was not available. Future studies may combine real purchase data with experimental data to determine a complete relationship of WTP and neonicotinoid pesticide use in production. Currently, the labeling of neonicotinoids on products is very limited; but, if neonicotinoid labeling becomes more prevalent (or mandatory) real purchasing data could be used to supplement and test the robustness of this study’s findings.

## Supporting information

S1 Appendix (DOCX)

S1 Fig Pollinator project research structure. (PDF)
