# Supplementary material for: Estimating willingness-to-pay for neonicotinoid-free plants: Incorporating pro-environmental behavior in hypothetical and non-hypothetical experiments
Source: PLoS One. 2021 May 20;16(5):e0251798. doi: 10.1371/journal.pone.0251798 (PMC8136652; doi:10.1371/journal.pone.0251798)
Supplement: S1 Appendix — (DOCX) [file pone.0251798.s001.docx]

# **S1 Appendix**

Table A1. Frequency distributions and corrected item-total correlations for the attitude scales

| Attitude toward neonicotinoid insecticides and pollinators | | Strongly disagree  (1) | disagree  (2) | Slightly disagree  (3) | Neither agree nor disagree (4) | Slightly  agree  (5) | Agree  (6) | Strongly agree (7) | $r_{i-t}$ |
| --- | --- | --- | --- | --- | --- | --- | --- | --- | --- |
| 1. Neonicotinoid pesticides are effective tools to protect plants from major and unwanted pests. | (*n*=420) | 6.4% | 9.8% | 11.9% | 34.1% | 16.4% | 11.7% | 9.8% | 0.11 |
|  | (*n*=74) | 2.7% | 5.3% | 5.3% | 48.0% | 20.0% | 13.3% | 5.3% | 0.01 |
| 2. I am concerned about the effects of neonicotinoid pesticides on pollinators. | (*n*=420) | 0.7% | 3.6% | 4.5% | 16.4% | 21.2% | 19.8% | 33.8% | **0.51** |
|  | (*n*=74) | 2.7% | 4.1% | 4.1% | 36.5% | 17.6% | 14.9% | 20.3% | **0.51** |
| 3. Use of neonicotinoid pesticides might be a cause of Colony Collapse Disorder (CCD) but I am not worried much about the extinction of bees and other pollinator insects. | (*n*=420) | 36.0% | 13.6% | 7.4% | 16.4% | 9.8% | 6.9% | 10.0% | 0.18 |
|  | (*n*=74) | 49.3% | 17.3% | 2.7% | 17.3% | 6.8% | 2.7% | 4.0% | 0.33 |
| 4. We may face a pollination crisis where crop yields decrease because of fewer pollinator insects. | (*n*=420) | 1.7% | 2.6% | 2.9% | 10.5% | 19.1% | 23.3% | 40.0% | **0.46** |
|  | (*n*=75) | 2.7% | 1.3% | 1.3% | 12.0% | 20.0% | 25.3% | 37.3% | **0.56** |
| 5. Pollination is vitally important to terrestrial ecosystems and to crop production. | (*n*=420) | 1.0% | 1.2% | 0.0% | 6.0% | 11.4% | 18.8% | 61.7% | **0.35** |
|  | (*n*=75) | 1.3% | 0.0% | 0.0% | 4.0% | 12.0% | 29.3% | 53.3% | **0.59** |
| 6. I would be willing to accept an increase in my annual taxes of $100 next year to promote neonicotinoid-free pesticides. | (*n*=420) | 17.9% | 11.0% | 8.6% | 21.7% | 15.0% | 10.7% | 15.2% | **0.22** |
|  | (*n*=73) | 12.3% | 13.7% | 12.3% | 31.5% | 11.0% | 8.2% | 11.0% | **0.54** |
| Attitude toward regulation of labeling neonicotinoids | | Strongly disagree  (1) | disagree  (2) | Slightly disagree  (3) | Neither agree nor disagree (4) | Slightly  agree  (5) | Agree  (6) | Strongly agree (7) | $r_{i-t}$ |
| 1. The federal government should require mandatory labeling of plants that are treated with neonicotinoid pesticides. | (*n*=420) | 2.4% | 1.4% | 1.7% | 13.8% | 12.9% | 18.3% | 50.0% | **0.55** |
|  | (*n*=75) | 1.3% | 0.0% | 4.0% | 17.3% | 10.7% | 16.0% | 50.1% | **0.77** |
| 2. Neonicotinoid labeling should be mandatory, because consumers have a right to be informed. | (*n*=420) | 1.0% | 0.7% | 1.2% | 9.8% | 13.3% | 18.8% | 55.2% | **0.62** |
|  | (*n*=75) | 0.0% | 2.7% | 0.0% | 16.0% | 10.7% | 18.7% | 52.0% | **0.76** |
| 3. Growers should be allowed to label plants treated with neonicotinoid on a voluntary basis. | (*n*=420) | 6.9% | 4.5% | 4.3% | 12.4% | 16.4% | 16.0% | 40.0% | 0.17 |
|  | (*n*=74) | 41.9% | 21.6% | 6.8% | 14.9% | 8.1% | 2.7% | 4.1% | 0.46 |
| Attitude toward the importance of information disclosed on a label |  | Very unimportant  (1) | Unimportant  (2) | Slightly unimportant  (3) | Neither important nor unimportant (4) | Slightly  important (5) | Important  (6) | Very important (7) | $r_{i-t}$ |
| 1. Pesticide free | (*n*=420) | 5.2% | 2.1% | 3.1% | 8.8% | 12.9% | 22.9% | 45% | **0.65** |
|  | (*n*=71) | 2.8% | 0.0% | 4.2% | 5.6% | 21.1% | 22.5% | 43.7% | **0.74** |
| 2. Neonicotinoid free | (*n*=420) | 4.1% | 2.9% | 3.3% | 36.0% | 15.2% | 12.9% | 25.7% | **0.70** |
|  | (*n*=75) | 2.7% | 2.7% | 2.7% | 22.7% | 16.0% | 20.0% | 33.3% | **0.66** |
| 3. Non-GMO/GMO free | (*n*=420) | 7.6% | 3.8% | 6.0% | 27.9% | 15.2% | 14.1% | 25.5% | **0.73** |
|  | (*n*=73) | 5.5% | 8.2% | 5.5% | 27.4% | 15.1% | 15.1% | 23.3% | **0.74** |
| 4. Certified Organic | (*n*=420) | 6.7% | 4.8% | 5.2% | 25.2% | 17.9% | 16.7% | 23.6% | **0.78** |
|  | (*n*=75) | 2.7% | 2.7% | 2.7% | 22.7% | 20.0% | 22.7% | 25.7% | **0.79** |
| 5. Organically produced | (*n*=420) | 6.4% | 4.3% | 5.0% | 22.9% | 22.1% | 18.3% | 21.0% | **0.79** |
|  | (*n*=75) | 4.1% | 2.7% | 2.7% | 20.3% | 21.6% | 25.7% | 23.0% | **0.80** |

Notes: Attitude scales with relatively higher corrected item-total correlations ($r_{i-t}$) in both studies (in bold) were selected to construct the attitude metrics.

Table A2. Principal components analysis of attitude items

|  | Study 1:  Online Choice Experiment | | | | Study 2:  Experimental Auction | | | | | |
| --- | --- | --- | --- | --- | --- | --- | --- | --- | --- | --- |
| Attitude toward neonicotinoid insects and pollinators | Factor 1 | Factor 2 | KMO Test | | Factor 1 | Factor 2 | | Factor 3 | KMO Test | |
| 1. Neonicotinoid pesticides are effective tools to protect plants from major and unwanted pests. |  | 0.78 | 0.49 | 0.64 |  |  | | 0.98 | 0.35 | 0.74 |
| 2. I am concerned about the effects of neonicotinoid pesticides on pollinators. | 0.78 |  | **0.68** |  | 0.77 |  | |  | **0.83** |  |
| 3. Use of neonicotinoid pesticides might be a cause of Colony Collapse Disorder (CCD) but I am not worried much about the extinction of bees and other pollinator insects. |  | 0.78 | 0.55 |  | -0.59 |  | |  | 0.70 |  |
| 4. We may face a pollination crisis where crop yields decrease because of fewer pollinator insects. | 0.80 |  | **0.67** |  |  | 0.96 | |  | **0.69** |  |
| 5. Pollination is vitally important to terrestrial ecosystems and to crop production. | 0.69 |  | **0.65** |  | 0.80 |  | |  | **0.73** |  |
| 6. I would be willing to accept an increase in my annual taxes of $100 next year to promote neonicotinoid-free pesticides. | 0.53 |  | **0.62** |  | 0.70 |  | |  | **0.81** |  |
| Attitude toward regulation of labeling neonicotinoids |  |  |  |  |  |  | | |  |  |
| 1. The federal government should require mandatory labeling of plants that are treated with neonicotinoid pesticides. | 0.90 |  | **0.51** | 0.51 | 0.92 |  |  | | **0.57** | 0.61 |
| 2. Neonicotinoid labeling should be mandatory, because consumers have a right to be informed. | 0.92 |  | **0.51** |  | 0.92 |  |  | | **0.57** |  |
| 3. Growers should be allowed to label plants treated with neonicotinoid on a voluntary basis. |  |  | **0.67** |  | -0.69 |  |  | | **0.91** |  |
| Attitude toward the importance of information disclosed on a label |  |  |  |  |  |  | | |  |  |
| 1. Pesticide free | 0.77 |  | **0.88** | 0.83 | 0.86 |  |  | | **0.78** | 0.78 |
| 2. Neonicotinoid free | 0.81 |  | **0.87** |  | 0.77 |  |  | | **0.74** |  |
| 3. Non-GMO/GMO free | 0.83 |  | **0.91** |  | 0.83 |  |  | | **0.91** |  |
| 4. Certified Organic | 0.87 |  | **0.78** |  | 0.89 |  |  | | **0.75** |  |
| 5. Organically produced | 0.88 |  | **0.78** |  | 0.89 |  | | | **0.77** |  |

Notes: Factors with eigenvalue larger than one are retained. Factors with loadings of 0.50 and below (in absolute value) are not reported in the table. Attitude scales with relatively higher KMO Test statistics in both studies (in bold) were selected to construct attitude metrics.
