# Supplementary figures and images for: Estimating willingness-to-pay for neonicotinoid-free plants: Incorporating pro-environmental behavior in hypothetical and non-hypothetical experiments
Source: PLoS One. 2021 May 20;16(5):e0251798. doi: 10.1371/journal.pone.0251798 (PMC8136652; doi:10.1371/journal.pone.0251798)

**Figure R1. Pollinator Project Research Structure**

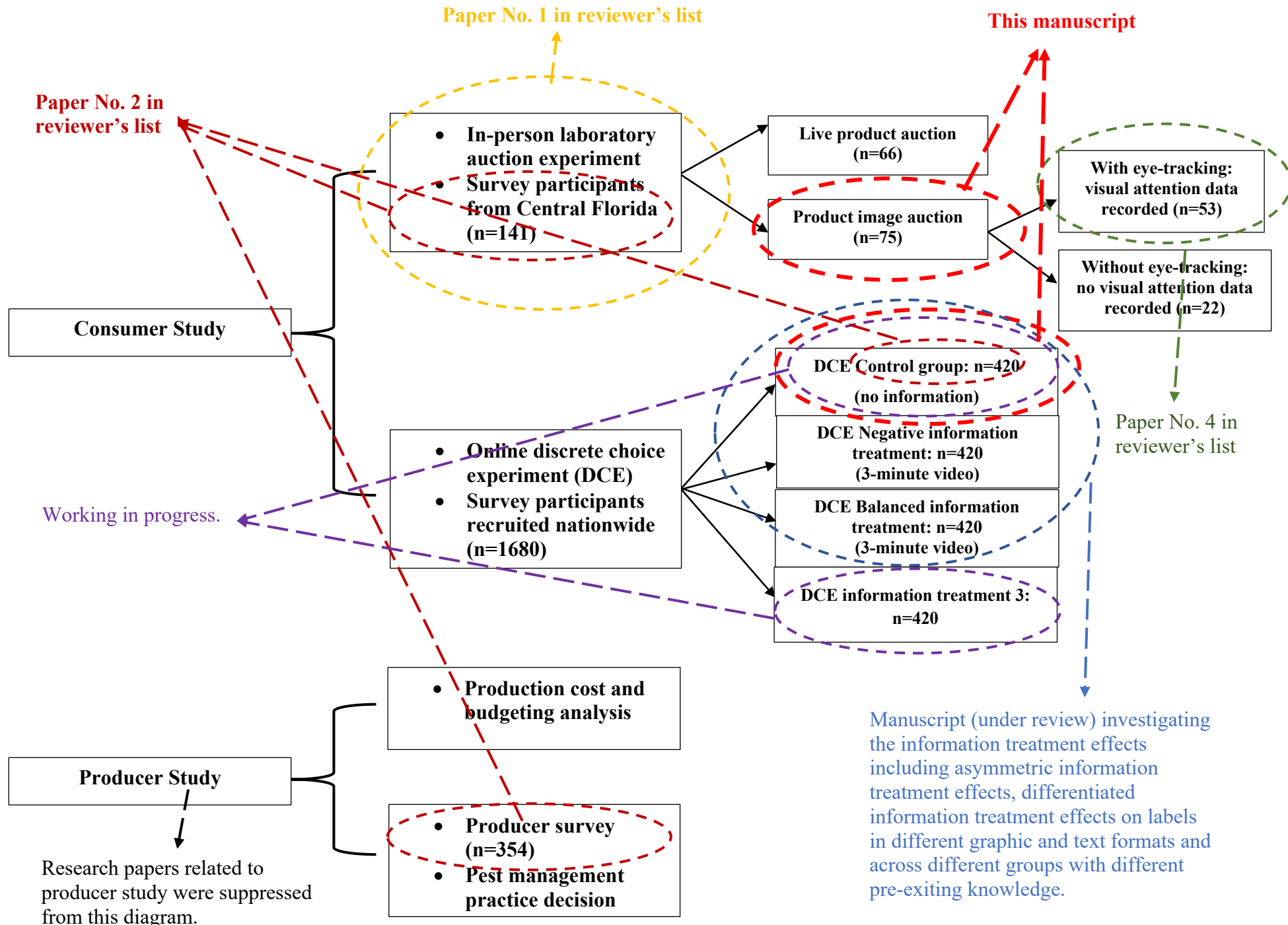

Supplement: S1 Fig — (PDF) [file pone.0251798.s002.pdf]
